# Migration, work, and retirement: the case of Mexican-origin populations

**DOI:** 10.1017/s1474747221000342

**Published:** 2021-09-09

**Authors:** Emma Aguila, Zeewan Lee, Rebeca Wong

**Affiliations:** 1 Sol Price School of Public Policy, University of Southern California, Los Angeles, USA; 2 Lee Kuan Yew School of Public Policy, National University of Singapore, Singapore, Singapore; 3 Preventive Medicine and Community Health, University of Texas Medical Branch at Galveston, Galveston, USA

**Keywords:** Retirement, Hispanics, social security, immigrant older adults

## Abstract

Mexico and the United States both face rapid population aging as well as older populations with high poverty rates. Among the most vulnerable populations of retirement age in either nation are Mexican immigrants to the United States. This work uses data from the U.S. Health and Retirement Study and the Mexican Health and Aging Study to assess retirement decisions among persons born in Mexico and working in either nation as well as such decisions by non-Hispanic Whites in the United States. Social security system incentives matter for the retirement of Mexican immigrants in the U.S. but not for return-migrants in Mexico.

The United States has high poverty rates among its elderly. Among those at least 66 years of age, poverty in the United States is ninth-highest among 40 nations analyzed by the Organisation for Economic Co-operation and Development (OECD, 2018a). Among older adults in the United States, poverty rates are especially high for racial minorities – for instance, 17.7% for Hispanics compared to 6.6% for non-Hispanic whites (Flores and Radford, 2017). As older Hispanic populations are growing more rapidly than older populations of other racial groups in the United States (United States Census Bureau, 2018), poverty may increase among the U.S. elderly in the coming decades.

One reason for the greater income insecurity of older Hispanic adults is the increase of income inequality in recent decades. Since 1970, the Hispanic median income has lagged behind that of non-Hispanic whites at low, middle, and high-income levels (Kochhar and Cilluffo, 2018). This growing gap may be due to a larger proportion of Hispanics working in low-paid jobs with fewer benefits, often because of their lower levels of educational attainment (Tali *et al*., 2018). U.S. Hispanics also have low levels of participation in employer-sponsored retirement plans, as not many of them work with employers that offer such benefits (Butrica and Johnson, 2010).

Given their low levels of readiness for retirement, what determines when Hispanic workers retire? Little research has been done regarding retirement determinants for low-income older adults. In this study, we analyze retirement determinants for Mexican-born immigrants in the United States and Mexicans who migrated to the United States but have returned to Mexico (return-migrants). Among U.S. Hispanics, 52.1% are of Mexican origin; among older Hispanics, the poverty rate for the foreign-born is nearly 1.5 times that for U.S.-born (Flores and Radford, 2017).

To assess the determinants of retirement for Mexican-born immigrants in the United States, we analyzed data from the 2012 and 2014 waves of the Health and Retirement Study (HRS). Most Mexican-born immigrants in our sample arrived in the United States when they were 15–25 years of age – that is, at the beginning of their working life – and had spent, on average, at least 37 years in the United States. To put the retirement behaviors of Mexican-born immigrants in perspective, we compared them to those of non-Hispanic whites. Next, to assess the determinants of retirement for Mexicans who migrated to the United States and returned to Mexico, we used the 2012 and 2015 waves of the Mexican Health and Aging Study (MHAS) and compared their determinants of retirement to those of native Mexicans who never migrated (non-migrants). The HRS and MHAS have similar questions on income, social security benefits, wealth, health, and health insurance, among other relevant outcomes for this study. We linked where possible HRS records with those of the U.S. Social Security Administration (SSA).

By using the HRS and MHAS, we can account for institutional determinants of retirement beyond social security systems. These include health insurance and private pensions (Gustman and Steinmeier, 2005; Blau and Gilleskie, 2006; Coile and Gruber, 2007; French and Jones, 2011) and socioeconomic characteristics and health (French, 2005; Shultz and Wang, 2007). We can account for differences in access to private pensions and, for those with access to pension plans, pension type. These include defined-benefit (DB) pension plans, whose benefits can be determined ahead of time, and defined-contribution (DC) plans, whose benefits depend on employer and employee contributions (Gruber and Wise, 1999; Shoven and Slavov, 2014). Each poses different incentives to workers. In particular, DB-plan holders are likely to retire as soon as they meet eligibility requirements, when the real value of their benefit is high. DC-plan holders are likely to delay retirement in order to increase their benefits by working additional years.^1^

We also consider how access to health insurance influences retirement decisions. In the United States, most individuals obtain insurance from their employers until they reach the Medicare eligibility age of 65 (Garber and Skinner, 2008). In 2010, 63% of firms provided employer-sponsored insurance to their employees, but only 40% of firms providing employer-sponsored insurance extended coverage to retired workers (Claxton *et al*., 2010). This may provide a strong disincentive to retire before 65 in the United States (French and Jones, 2011). It also presents a stark contrast to the universal *Seguro Popular* health insurance program offered to all who lack institutional health insurance in Mexico (Parker *et al*., 2018). Finally, health itself can affect retirement decisions. Individuals with poor health are not as productive as those with good health. This affects earning trajectories that, in turn, affect retirement decisions (Currie and Madrian, 1999; Dwyer and Mitchell, 1999; Shultz and Wang, 2007).

To analyze the differing determinants of retirement across the groups, we constructed retirement conditional probit models using sociodemographic, health, health care utilization, health insurance, private pension, and social security system covariates. We estimated social security wealth for Mexican-born immigrants and non-Hispanic whites. We also estimated a forward-looking retirement incentive measure, the *peak value* of social security wealth (Coile and Gruber, 2007; Liebman *et al*., 2009), which compared the time when social security wealth reaches its maximum expected value with its present expected value. We found that the social security systems were an important determinant for retirement for Mexican-born immigrants in the United States, but that they mattered little for return-migrants in Mexico.

Previous studies have analyzed how immigrants fare relative to the native-born in social security benefits and other sources of retirement income (e.g., Gustman and Steinmeier, 2000; Sevak and Schmidt, 2014), as well as in retirement and social security claiming patterns (Borjas, 2011; Mudrazija *et al*., 2017; Lopez and Slalov, 2020). Many of these studies, however, did not differentiate by ethnicity among the foreign-born or by legal status, and they focused on a different question than determinants of retirement. Our paper contributes to the literature by exploring drivers of retirement among low-income immigrants. In our study, as we review later in our Discussion section, we are unable to differentiate immigrants by their U.S. documentation status.

We organize our study as follows. Section 1 describes the institutional arrangements of retirement in the United States and Mexico. Section 2 presents the data, outcome variables, and covariates that we use in our research. It also provides descriptive statistics of persons in our sample, their social security wealth, and peak value retirement incentive measures. Section 3 describes our empirical methodology, including the probit equations we use. Section 4 presents our results for the determinants of retirement for Mexican-born immigrants and non-Hispanic whites in the United States and Mexican-origin populations in Mexico. Section 5 provides a discussion of our results and Section 6 summarizes our findings and policy recommendations.

## 1. Institutional background

### 1.1. United States

The two main programs of the U.S. SSA are the Old Age and Survivors Insurance (OASI) program and the Disability Insurance program. In this article, we focus on OASI program benefits, which we refer to as social security benefits. Most individuals, including the self-employed, are required by law to contribute to social security via payroll or self-employment taxes (Zissimopoulos *et al*., 2007; AARP, 2021).^2^ All who have worked for at least 40 three-month quarters are eligible for social security benefits. Individuals can claim these benefits once they reach the early retirement age (ERA), but with benefits reduced for each month of retirement before the full retirement age (FRA). Those who work past the FRA see their benefits increase for each extra month of work until reaching age 70 (U.S. Social Security Administration, 2019a, 2019b). Social security benefits are provided monthly until death.

The U.S. social security system imposes different rules of eligibility (i.e., different ERA and FRA) and benefits calculation by age.^3^ Beneficiaries in different cohorts face different eligibility threshold ages. The original FRA was 65 for those born before 1938. A 1961 law implemented the ERA of 62, specifying benefits at that age would be 80% of the full benefit amount. A 1983 law raised the FRA to 66 for persons born between 1943 and 1954 and to 67 for those born in 1960 or later. For individuals born between 1938 and 1955, the FRA increased by 2 months from age 65 (i.e., 65 and 2 months for those born in 1938, 65 and 4 months for those born in 1939, and so on). Once the FRA reaches 67, those retiring at age 65 will receive 87% of the full benefit, and those retiring at age 62 will receive 70% of the full benefit (National Academy of Social Insurance, 2017; Social Security Administration, n.d.a, n.d.b).

For our analyses, we tailor the eligibility thresholds (i.e., ERA and FRA) and benefit rates of different age groups, or cohorts, in our HRS sample to the social security system rules for each cohort. We discuss in our methods section more details on how we calculated monthly benefits.

### 1.2 Mexico

One of the distinguishing characteristics of the labor market in Mexico is the large proportion of workers – 42% – in the informal sector.^4^ This has a sizeable effect on who is covered by the Mexican social security system. In contrast to the United States, Mexican self-employed workers are not required to contribute to the social security system.

Private sector workers are covered by the Mexican Institute for Social Security (Instituto Mexicano del Seguro Social, or IMSS), while public sector employees are covered by the Institute for Social Security and Services for State Workers (Instituto de Seguridad y Servicios Sociales de los Trabajadores del Estado, or ISSSTE). These institutes, which cover most of the formal labor force, provide retirees with social security benefits, health care, and other services. Other formal sector workers are covered by the Mexican Oil Enterprise (PEMEX), the Army, the Marines, or by private firms with their own social security and health care systems (Parker and Wong, 1997).

Informal sector workers in Mexico are currently entitled to a non-contributory pension or social pension at age 65 (Secretaría de Bienestar, 2019). This program is similar to the U.S. Supplemental Security Income (SSI) program. The Mexican non-contributory pension program age eligibility rules, geographic coverage, and means-testing rules have varied over time. For our period of analysis, the Mexican non-contributory pension program was available in urban and rural areas for adults 70 or older in 2012 and for those 65 or older from 2013 to 2018, excluding those who received social security benefits (Aguila and Angel, 2021). In 2012, the monthly benefit amount was $500 MXN or $72.90 in USD purchasing power parity (PPP); from 2014 to 2018, it was $580 MXN or $78.1 in USD PPP (Aguila *et al*., 2016). Informal sector workers are also entitled to universal health care services provided by the *Seguro Popular* program (Parker *et al*., 2018). In 2017, *Seguro Popular* covered 39.3% of the Mexican population, IMSS 36.3%, and ISSSTE 5.6%, with the remaining 18.8% covered by other private or public institutions (INEGI, 2018).

## 2. Data and descriptive results

### 2.1 Data sources

Our principal data sources, as noted, are the 2012 and 2014 waves of the U.S. HRS and the 2012 and 2015 waves of the MHAS. In both, we restricted our sample to individuals 50–80 years of age.

The HRS is a nationally representative biennial panel survey conducted every 2 years since 1992 of U.S. individuals at least 50 years of age and their spouses. It includes an oversample of Hispanics in general and of Mexican-Americans specifically (Ofstedal and Weir, 2011). The core HRS interview is offered in English or Spanish.

We link, where possible, HRS respondents to their past earnings records available through the SSA data.^5^ For such individuals, we used social security earnings records to calculate social security wealth. For HRS respondents without HRS-SSA linkages – as well as for HRS respondents with linkages whose labor status was shown as working but whose earnings were zero or negative – we imputed past earnings as described in the Appendix.

Among HRS respondents in 2012, 841 were Mexican-born and 11,914 were non-Hispanic white. Among the 841 Mexican-born individuals, 429 had their earnings history through HRS-SSA linkages, and we imputed earnings for 412. Among the 11,914 non-Hispanic whites, 9,171 had earnings history through HRS-SSA linkages, and we imputed past earnings for 2,743. After excluding from our sample those who were outside the age bracket of 50–80 (Mexican-born: 49 or 5.8%; non-Hispanic white: 2,305 or 19.3%), who were not working in 2012 (Mexican-born: 418 or 49.7%; non-Hispanic white: 5,499 or 46.2%), who had no follow-up in 2014 (Mexican-born: 26 or 3.1%; non-Hispanic white: 306 or 2.6%), or who had missing covariates (Mexican-born: 37 or 4.4%; non-Hispanic white: 156 or 1.3%), our HRS sample consisted of 311 Mexican-born and 3,648 non-Hispanic white respondents (see Appendix for more detailed information).

Our sample size for Mexican-born immigrants was smaller than for non-Hispanic whites. The extent of attrition from non-response was similar among the two groups and represented only a small proportion of the respondents dropped from the sample. To confirm the representativeness of our Mexican-born HRS sample, we compared our sample to data from the 2012 American Community Survey (ACS). We found no statistically significant differences between our HRS sample and the ACS sample of native Mexicans in household size or educational attainment (see Table A1 of online Appendix). Real monthly salary income was about $100 higher for the HRS sample, but the difference was only marginally significant. Our HRS sample was more likely to be male and younger than the comparable ACS sample, but the differences, while statistically significant, were not great (e.g., average age of 57.9 years for our HRS sample and 59.9 years for the ACS sample). While there was a more substantial difference in the proportion of respondents who were in couples (77% for our HRS sample and 69% for the comparable ACS sample), overall the ACS and our HRS sample of Mexican-born residents of the United States 50–80 years of age were more similar to each other than they were to non-Hispanic whites. We also conducted additional robustness analysis of our regression estimates for Mexican-born immigrants by adding more HRS waves (2000–2014). We discuss this further in the Results section. One limitation to our analysis is that foreign-born respondents in the HRS did not provide their documentation status. As a result, we were unable to identify undocumented Mexican-born immigrants. We consider in the Discussion section how this may affect our analysis.

The MHAS is a nationally representative panel study, conducted in 2001, 2003, 2012, 2015, and 2018, of Mexican residents born before 1951 as well as their spouses regardless of age. The MHAS oversamples Mexican states with high numbers of migrants to the United States. The MHAS questionnaire is based on that of the HRS, and the phrasing of questions in both surveys is comparable (Soldo *et al*., 2002).

Our analytic sample included MHAS respondents who migrated to the United States and returned to Mexico (return-migrants), as well as those who remained in Mexico (non-migrants). Among MHAS respondents in 2012, 1,562 were return-migrants and 14,161 were non-migrants. After excluding those who were outside the age bracket of 50–80 (return migrant: 233 or 14.9%; non-migrant: 1,871 or13.2%), who were not working in 2012 (return migrant: 665 or 42.6%; non-migrant: 7,463 or 52.7%), who had no follow-up in 2015 (return migrant: 87 or 5.6%; non-migrant: 628 or 4.4%), or who had missing covariates (return migrant: 45 or 2.9%; non-migrant: 209 or 1.5%), our sample included 532 return-migrant respondents and 3,990 non-migrant respondents.^6^ We provide a detailed description of our samples in the Appendix.

### 2.2 Outcome variable

Our outcome variable is a binary retirement indicator defined as follows:

(1) $$R_{t}=\{\begin{array}{c} 0\text{ if working in }t\text{ and }t+1\\ 1\text{ if working in }t\text{ and fully retired in }t+1 \end{array}.$$

In other words, we compared those (coded 0) who worked in both waves of the surveys with those (coded 1) who worked in the first wave and retired by the second wave of the surveys. We excluded from our analysis those who reported being disabled, unemployed, or not in the labor force and included only those fully retired.

### 2.3 Covariates

In our analysis, covariates are in time *t*. HRS and MHAS covariates in our analysis include birth cohorts (cohorts born before 1941, between 1942–1947, 1948–1953, and 1954–1959), gender (1 = male, 0 = female), number of years of education, whether the respondent was part of a couple (1 = yes, 0 = no), number of household members, U.S. citizenship or permanent residency eligibility, logarithm of monthly salary income, and net wealth. We defined net wealth as the amount of money in checking and savings accounts, bonds, stocks, deposits, mutual funds, primary and secondary housing, and any other savings less debts. To account for discrepancies between the United States and Mexico in purchasing power, we converted all income, wealth, and expenditure variables in MHAS to 2012 U.S. dollars PPP (OECD, 2018b).

We also included covariates on physical and mental health. We measured physical health with a binary indicator on whether individuals had any of the following chronic health conditions, as diagnosed by a health care provider: high blood pressure or hypertension; diabetes or high blood sugar; cancer or a malignant tumor; chronic lung disease; heart attack, coronary heart disease, angina, congestive heart failure, or other heart problems; and stroke. We measured respondents’ mental health with a modified CES-D depressive symptoms count (0–8 scale), based on the self-reported frequency of experiencing in the past week feeling depressed, feeling that everything was an effort, restless sleep, feeling unhappy, feeling alone, not enjoying life, feeling sad, or feeling tired (Turvey *et al*., 1999; Aguilar-Navarro *et al*., 2007).

For healthcare insurance and expenditures in the United States, we included a Medicare eligibility indicator (1 if aged 65 and above, 0 if not), a dummy variable indicating access to employer-sponsored health insurance during working years (1 = yes, 0 = no), and the logarithm of annual out-of-pocket health expenditures.^7^ For Mexico, we included a covariate for coverage of health insurance in the Mexican social security systems (1 = yes, 0 = no), affiliation to the public health insurance program *Seguro Popular* (1 = yes, 1 = no), and the logarithm of annual out-of-pocket health expenditures.

For MHAS respondents, we included an identifier for eligibility to receive the non-contributory pension (1 = aged 65 and above, 0 = not). For HRS respondents, we added an identifier for respondents’ contribution to private pension by plan type (DB plans, defined contribution plans, both, or none). As noted earlier, defined benefit plans provide stronger incentives for early retirement than DC plans do (Gruber and Wise, 1999). In Mexico, less than 10% of firms provide private pensions to employees (Aguila, 2014). In our sample, less than 1% of MHAS respondents receive private pensions; we therefore excluded this variable from the MHAS analysis.

### 2.4 Social security covariates

U.S. social security benefits are typically determined by individual earnings – the 35 years of highest annual earnings – and age at retirement (U.S. Social Security Administration, 2019a, 2019b). Once we obtained past earnings for our sample, whether from HRS-SSA linkages or our imputations, we adjusted them using National Average Wage Indices (Social Security Administration, 2019a, 2019b). For simplicity, we excluded any cost-of-living adjustments to which recipients are entitled. We then used the best 35 years in each individual’s earnings history to calculate the average indexed monthly earnings (AIME).

Once we calculated the AIME for each respondent, we applied a non-linear function to estimate social security benefits – also known as the Primary Insurance Amount (PIA).^8^ The formula estimates an individual’s PIA by summing three separate percentages of portions of the AIME predetermined by the SSA.^9^ Based on the PIA, we calculated the social security benefits for each possible retirement age from current year *t* to the year in which a respondent would turn 120, taking into consideration the difference between each respondent’s retirement age and the FRA, and also accounting for mortality as shown in the equation below. To project future earnings (2015 and beyond in the HRS), we set the earnings of all to increase by 1% every year. While both the benefit and tax rates of the OASI program influence individual retirement decisions (Fields and Mitchell, 1984; Coile and Gruber, 2007), we did not consider tax rates in this study in order to simplify our analysis. We also did not consider spouse or dependent benefits for two reasons. First, we lacked information on them. Second, previous research showed such benefits have little effect on the primary earner’s labor supply decisions (Knapp, 2014).^10^

We calculated social security wealth (SSW_*t*_) as the expected net present value of a worker’s social security benefits to be received until death if retiring at age *t*, as follows:

(2) $${\text{SSW}}_{t}=\overset{T}{\underset{s=R}{\sum }}\frac{\left[pr_{s|t}\times ssb_{s}\right]}{{\left(1+d\right)}^{\left(s-t\right)}},$$

where *pr*_*s*|*t*_ is the probability the individual is alive at time *s* conditional on being alive at time *t*. *ssb*_*s*_ is the social security benefit to be collected on a monthly basis if the individual chooses to retire at time *s* – also known as the PIA in the United States. Following Coile and Gruber (2007), we set *d*, the real discount rate, to be 3% and *T*, the maximum possible age reachable by an individual, equal to age 120 according to U.S. life-expectancy tables (Arias *et al*., 2016).^11^ *R* is the age of retirement. We computed the survival probabilities (*pr*_*s*|*t*_) as $pr_{\text{s}|\text{t}}=\overset{s-1}{\underset{t}{\prod }}(1-\lambda_{t})$, whereby *λ*_*t*_ is a hazard function where *λ*_*t*_ = *d*_*t*_/*S*_*t*_. Here, *d*_*t*_ is the number of people dying in period *t*, and *S*_*t*_ is the number of survivors at time *t*. We obtained information on survival and mortality prospects for the computation of *pr*_*s*|*t*_ from life-expectancy tables published by the U.S. Centers for Disease Control and Prevention. To account for significant differences in survival probabilities by demographic characteristics, we applied different survival probabilities by gender and race – generating distinct probabilities for male-Mexican-born respondents, female-Mexican-born respondents, male-non-Hispanic whites, and female-non-Hispanic whites.

We computed social security wealth to be non-zero for individuals starting at age 60 and increasing afterward. Although the ERA is 62, this was consistent with previous research that accounts for cases where individuals retire before the ERA (e.g., age 60) but delay claiming benefits until they reach the ERA (Coile and Gruber, 2007).^12^

For MHAS respondents, we included an indicator for whether respondents contributed to social security in Mexico (1 = yes, 0 = no), and one for whether they contributed to social security in the United States (1 = yes, 0 = no).

Next, we computed peak-value retirement incentives by comparing two points in time. These are (1) the future point when social security wealth is at its maximum expected value and (2) the current point when social security wealth is calculated based on monthly benefits to be received with immediate retirement. That is, we calculated the peak-value retirement incentives by taking the difference between (1) the maximum expected social security wealth one would have by delaying retirement to the optimal year, and (2) the social security wealth one would have if retiring immediately. The peak value is included in the HRS estimation models.

Introduced by Coile and Gruber (2000), the peak value is a simplified metric of the retirement incentives imposed by the social security wealth accumulation that stems from the option value (Lumsdaine *et al*., 1992; Ausink and Wise, 1996).^13^ The peak value effectively captures the retirement incentives embedded in social security wealth accumulation without imposing as many assumptions as the option value does (Stock and Wise, 1990). As the peak value does not include labor earnings, it provides more accurate estimates of the effect of social security retirement incentives on the probability of retirement (Coile and Gruber, 2007). By controlling separately for individual earnings and using SSA rules to identify the main source of variations in social security wealth accumulation, we may eliminate the key differences in the peak value and the option value measures (Samwick, 2000; Friedberg and Webb, 2005).

### 2.5 Descriptive results

Table 1 summarizes the characteristics for the four groups we compare: HRS samples of (1) non-Hispanic whites, and (2) Mexican-born immigrants, and MHAS samples of (3) Mexicans who did not migrate to the United States, and (4) Mexicans who migrated to the United States but returned to Mexico.

We observed the largest proportion of males among return-migrants in Mexico and the smallest proportion of males among non-Hispanic whites. MHAS respondents, both non-migrants and return-migrants, and Mexican-born immigrants in the MHAS had similar levels of education, which were about half the level of education of non-Hispanic whites. Return-migrants in MHAS were most likely to be in couples, while Mexican-born immigrants in the HRS had the largest average household size. Mexican-born immigrants in the HRS had higher monthly salary income than the MHAS groups but lower salary income than non-Hispanic whites. Mexican-born immigrants had the lowest net wealth among all groups. Among the MHAS return-migrants, only 1.3% were U.S. citizens or had the right to permanently reside in the United States (we did not have this information for Mexican-born immigrants).

All three Mexican-origin groups were less likely to have chronic conditions than non-Hispanic whites. While Mexican-born immigrants were less likely to experience depressive symptoms than the other Mexican groups, they reported more depressive symptoms than non-Hispanic white respondents. Non-Hispanic whites generally had more health insurance coverage – either from an employer or Medicare – than Mexican-born immigrants did. The sharp difference in Medicare eligibility −33.0% for non-Hispanic whites but only 8.4% for Mexican-born immigrants – was likely because Mexican-born immigrants in our sample were younger than non-Hispanic whites.

Among our MHAS respondents, non-migrants were more likely to have social security health insurance while return-migrants were more likely to have *Seguro Popular*. MHAS respondents reported far fewer out-of-pocket health care expenditures than HRS respondents do.

Among MHAS respondents, a higher proportion of return-migrants than non-migrants were eligible to collect the non-contributory pension. This may reflect the impact of the means-testing rule of the program in 2015, under which those who collected social security benefits were ineligible for the non-contributory pension. This is also not surprising given return-migrants are older and hence likely to have reached eligibility age for the pension. In the United States, non-Hispanic white respondents are more likely to have access to both defined benefit and defined contribution pension plans than do Mexican-born respondents.

Table 2 shows median social security wealth and peak values in the HRS samples. Percentiles were computed separately for each group. For both Mexican-born immigrants and non-Hispanic whites, social security wealth peaked around ages 68–69 and decreased afterwards. Such patterns are consistent with those in previous research (Coile and Gruber, 2007). Up to age 66, social security wealth was larger for non-Hispanic whites than for the Mexican-born immigrants. From age 66 onwards, social security wealth was slightly higher for Mexican-born immigrants. This is because survival probabilities for Hispanics are higher than for non-Hispanic whites, which reduces the social security wealth gap between non-Hispanic whites and Mexican-born immigrants.

Both groups of HRS respondents saw their peak value incentive at its highest level at age 60–61 and then decrease each year, turning negative at age 69 for both groups.

**Figure 1.**
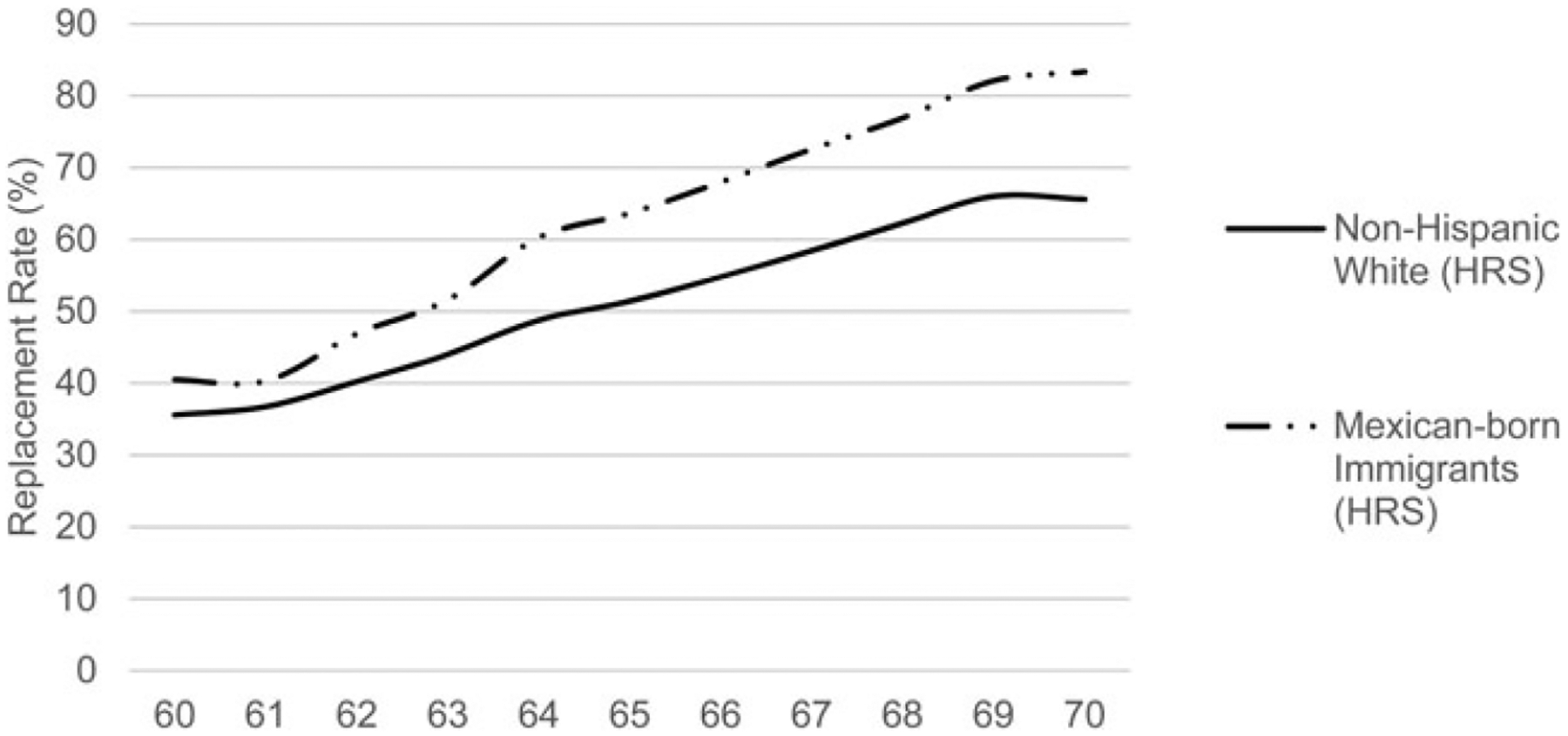
Replacement rate for the 50th percentile. *Source*: author’s calculations.

Figure 1 shows the median replacement rates of social security benefits. Replacement rates were calculated by dividing monthly social security income – the monthly benefits to be collected by retiring at each age, with the delayed and early claiming rates implemented – by total monthly income.^14^ The higher the rate, the greater the monthly benefits one receives by retiring rather than working at each age. The replacement rate can be interpreted as an alternative retirement incentive measure to the peak value. For non-Hispanic whites and Mexican-born immigrant respondents, replacement rates grew with age until age 69. This suggests that they both experienced stronger retirement incentives each year until age 69. Replacement rates were higher for Mexican-born immigrants than for non-Hispanic whites.

## 3. Empirical methods

To assess the relative contributions of different covariates to retirement decisions between *t* and *t* + 1, we estimated for individuals who were working in period *t* the following retirement conditional probit model:

(3) $$\text{Pr}({\text{Retirement}}_{i}=1)=f(\beta_{0}+\beta_{1}SSW_{i}+\beta_{2}PV_{i}+\beta_{3}X_{i}+\beta_{4}H_{i}+\beta_{5}HC_{i}+\beta_{6}PP_{i}),$$

where *Retirement*_*i*_ indicates with value 1 when the individual transitions from working in the first wave to retirement in the second wave and 0 when the individual continues working across waves. Covariates are in time *t*. *SSW*_*i*_ is social security wealth, *PV*_*i*_ is the peak value social security wealth incentive measure, *X*_*i*_ is a matrix that includes sociodemographic characteristics, *H*_*i*_ are physical and mental health variables, *HC*_*i*_ are health care utilization variables, and *PP*_*i*_includes an indicator of contributing or receiving private pensions. We clustered standard errors at the household level. We estimated this specification for Mexican-born immigrants and non-Hispanic whites in HRS.

Next, to better understand retirement patterns of formal and informal sector workers in Mexico, we estimated a retirement conditional probit model of retirement between *t* and *t* + 1, including only non-migrants or return-migrants in Mexico who were working in period *t*:

(4) $$\text{Pr}({\text{Retirement}}_{i}=1)=f(\beta_{0}+\beta_{1}X_{i}+\beta_{2}H_{i}+\beta_{3}HC_{i}+\beta_{4}NCP_{i}+\beta_{5}SSC_{i}),$$

where *Retirement*_*i*_, *X*_*i*_, *H*_*i*_, and *HC*_*i*_ are as above, while here *NCP*_*i*_ indicates whether the respondents were eligible to the Mexican non-contributory pension and *SSC*_*i*_ indicates whether respondents contributed to the Mexican or U.S. social security systems. We again clustered standard errors at the household level.

## 4. Results

Table 3 shows the marginal effects of the probit model regressions as described in Equation (3) for our original sample and Table 4 displays additional robustness analysis using additional HRS waves from 2000 to 2014. Table 5 displays the marginal effects of the probit model regressions among the non-migrants and return-migrants in MHAS, as detailed in Equation (4). For all models, the outcome variable is a binary indicator for retirement, which takes a value of 1 if working in 2012 and retiring in the second wave, and a value of 0 if working in both waves. In Tables 3 and 4, Specification I, we ran the model with only social security wealth and the peak value incentive measure as regressors. The model for Specification II includes social security wealth, the peak value incentive measure, and sociodemographic covariates. Specification III includes social security wealth, peak value, and sociodemographic covariates, as well as health, health care utilization, and private pension covariates.^15^

In Specification I in Table 3, at the sample mean of all regressors, a $10,000 increase in social security wealth increased retirement likelihood by 1.1 percentage points for non-Hispanic whites (6.96% from the sample average retirement likelihood of 15.8%) and 1.3 percentage points for Mexican-born immigrants (18.31% from the sample average retirement likelihood of 7.1%). At the sample mean of all regressors, a $10,000 increase in the peak value incentive reduced retirement likelihood by 3.9 percentage points for non-Hispanic whites (−24.68% from the sample average retirement likelihood of 15.8%) and 1.8 percentage points for Mexican-born immigrants (−25.35% from the sample average retirement likelihood of 7.1%). The positive coefficient for social security wealth and negative coefficient for the peak value incentive measure was consistent with previous literature (Gruber and Wise, 2004). Higher social security wealth implies greater financial security, which can induce retirement. A higher peak value incentive measure suggests that social security wealth that may be claimed later is greater than that which may be claimed today and is likely to induce workers to delay retirement (Coile and Gruber, 2007; Lusardi and Mitchell, 2007).

When we add our sociodemographic covariates in Specification II, the sign and magnitude of the social security wealth coefficients remained similar to those of specification I. However, the peak value incentive measure was no longer statistically significant for both groups. The older cohort was more likely to retire than the younger one, but this effect was not statistically significant for Mexican-born immigrants. The increases in monthly income decreased the likelihood of retirement for non-Hispanic whites and Mexican-born immigrants.

Next, we show the results from Specification III where we add to the previous specification the covariates on health, health care utilization, and private pension. Adding these covariates shows a $10,000 increase in social security wealth increased the probability of retirement for non-Hispanic whites by 0.5 percentage points (3.16% from the sample average retirement likelihood of 15.8%) and for Mexican-born immigrants by 0.9 percentage points (12.67% from the sample average retirement likelihood of 7.1%). Under this specification, the peak value variable became statistically insignificant for the two HRS groups. Chronic conditions increased retirement chances by 3.6 percentage points for non-Hispanic whites and 4.9 percentage points for Mexican-born immigrants. Annual out-of-pocket health expenditures did not have a significant effect on retirement for either HRS group.

We conducted additional robustness tests for this specification including Waves 5–12 from HRS (2000–2014), finding for the most part similar results in terms of sign and statistical significance (see Table 4). With the increased sample size, the peak value incentive measure was statistically significant for both groups in Specification II. In Table 3, we found no association between Medicare eligibility and retirement decisions for both groups, although the association was statistically significant when we ran the analysis using the 2000–2014 HRS waves for non-Hispanic white. Expectation of future access to private pension benefits also had a significant effect on retirement among non-Hispanic whites and Mexican-born immigrants. Having defined contribution plans relative to having defined benefit plans reduced retirement for both groups. The finding that having a defined contribution plan reduces the likelihood of retirement was consistent with previous research (e.g., Shoven and Slavov, 2014).

It is worth noting that Mexican-born immigrants had different determinants of retirement than do non-Hispanic whites. The findings for non-Hispanic whites were consistent with previous research: those with less education, lower salary income, worse physical health, access to Medicare, lack of employer-sponsored health insurance, and having a defined benefit plan were more likely to retire (Karpanzalo *et al*., 2005; Clark *et al*., 2006; French and Jones, 2011). The positive influence of access to Medicare eligibility and the negative association of employer-sponsored health insurance on retirement decisions is particularly noteworthy. Given that employer-sponsored health insurance tends to create a sizeable ‘job lock’, whereby individuals delay retirement to preserve their insurance coverage, the primary role of Medicare on retirement decisions appears to lie in its ability to lift the job lock (French and Jones, 2011). Among Mexican-born immigrants, those with physical health conditions were more likely to retire, and those with defined contribution private pensions relative to those with a defined benefit plan were less likely to retire. In contrast to non-Hispanic whites, for Mexican-born immigrants being older or having more education, or Medicare eligibility did not change the likelihood of retirement.

Table 5 shows the determinants of retirement for non-migrant and return-migrant respondents in the MHAS. Among both groups, males were less likely to retire, while those in larger households were more likely to retire. The positive influence of household size on retirement decisions may be related to family transfers and pooling of household resources, allowing older adults without social security access to retire (DeGraff *et al*., 2018). Salary income affects retirement for non-migrants but not return-migrants.

Both physical and mental health were significant determinants of retirement among non-migrants but not return-migrants. Health insurance, eligibility for a non-contributory pension, and social security contributions were also significant determinants of retirement for non-migrants but not return-migrants. In short, return-migrants seem less responsive to these determinants of retirement compared to non-migrants.

## 5. Discussion

We found that Mexican-born immigrants and non-Hispanic whites had similar responses to social security incentives, matching those in earlier research in the United States and other European countries (Gruber and Wise, 1999, 2004). Mexican-born immigrants, on average, had lower annual earnings than non-Hispanic whites for each year but a higher average peak value. This could be a result of Mexican-born immigrants having worked more continuously and earning non-zero earnings for longer than non-Hispanic whites. Due to the progressivity of social security, the gains from continued work can be greater for those with a lower earnings history – eventually leading to the greater average peak value of Mexican-born immigrants. Our measure of social security wealth was similar for Mexican-born immigrants and non-Hispanic whites. This is because of the higher replacement (shown in Figure 1) for Mexican-born immigrants, resulting from the redistributive component of social security payments and higher survival probabilities for Hispanics. The findings of Sevak and Schmidt (2014), who used the HRS to document differences in social security benefits between foreign-born and native-born, match ours. They found that the foreign-born who had been in the United States for longer periods of time had similar or higher monthly benefits than the native born, while foreign-born persons who arrived in the United States closer to retirement age had fewer quarters of social security covered earnings and lower monthly benefits.

Our finding that Mexican-born immigrants and non-Hispanic whites react similarly to social security incentives suggests there were other contributors to the relatively high rates of poverty among older Mexican-born immigrants. Munnell *et al*. (2018) found that 48% of non-Hispanic white households headed by persons 30–59 years of age were ‘at risk’ for financial insecurity in old age, compared to 61% of Hispanic households. The authors found a larger wealth and income gap between non-Hispanic whites and Hispanics *before retirement* than *after retirement*. The authors suggested that this can be explained by the SSA’s progressive formula increasing social security wealth of low-income individuals in the post-retirement years, reducing the gap between non-Hispanic whites and Hispanics. Our analysis is supported by Hou and Sanzenbacher (2020), who found that social security wealth provides, on average, a 95% replacement rate (ratio between social security wealth and pre-retirement income) for Hispanic households.

Hou and Sanzenbacher (2020) found other types of wealth, such as employer-provided pensions and financial and housing wealth, were more unequal for non-Hispanic whites and Hispanics after retirement, and that this led to financial insecurity in old age for Hispanics. Dettling *et al*. (2017) found that non-Hispanic white homeowners held more home equity than Hispanics and had more diversified asset portfolios, including business ownership, private retirement accounts, and financial savings (stocks, bonds, and mutual funds). Furthermore, a larger proportion of Hispanics than non-Hispanic whites had previously been denied credit, with such credit constraints leading them to accumulate wealth at a lower rate than non-Hispanic whites do. In 2016, the unbanked rate was 20% for Hispanic households and 4% for non-Hispanic white households (Federal Deposit Insurance Corporation, 2017). Blanco *et al*. (2018) reported that middle-aged and older Hispanics were less likely to own a bank account than non-Hispanic whites. This difference was explained mostly by language barriers for Hispanics. Brown (2009) also found that the lower socioeconomic status and poor living conditions of Hispanics, coupled with adverse effects of racism and residential segregation, may limit their access to better jobs and contribute to poor working conditions throughout their working life, reducing their chances to accumulate wealth and prepare for retirement.

Mexican-born immigrants in our sample had been in the United States for 37 years on average. Our sample of Mexican-born immigrants was primarily individuals who arrived when they were 15–25 years old, or near the beginning of their working life. Mexican-born immigrants in our study also had much lower levels of education than non-Hispanic whites. As a result, our findings differed from a number of previous studies (e.g., Gustman and Steinmeier, 2000; Borjas, 2011) that either focused on migrants arriving at older ages or did not analyze the heterogeneity of immigrants by race/ethnic backgrounds.^16^ Our results differed because we focused specifically on Mexican-born immigrants. Our findings aligned more with Sevak and Schmidt (2014), who analyzed immigrants by demographic and socioeconomic characteristics, including their number of years in the United States.

Among our HRS respondents, while higher levels of education reduced the likelihood of retirement for non-Hispanic whites, lower levels of education did not appear to increase it for Mexican-born immigrants. Higher education also did not influence retirement for non-migrants and return-migrants. Access to private pensions was low for Mexican-born immigrants, but those with such pensions were less likely to retire. For Mexican-born immigrants, Medicare eligibility also did not affect retirement.

For both MHAS groups, greater household size increased the likelihood of retirement, but household size did not affect retirement for U.S. Mexican-born immigrants. In Mexico, higher salary income decreased the likelihood of retirement for non-migrants but not for return-migrants. Non-migrants with chronic conditions, depressive symptoms, health insurance, eligibility for a non-contributory pension, or social security contributions in Mexico were more likely to retire than others. For return-migrants, social security system contributions and non-contributory pension availability did not affect retirement decisions. This may be a result of the inability of Mexican return-migrants to access social security benefits in either the United States or Mexico. Indeed, only 5% of return-migrants in Mexico who contributed to the U.S. social security system expect to receive benefits from it (Aguila and Vega, 2017).

Our work has some possible limitations. Previous research has found that Mexican-born immigrants are a self-selected population (e.g., Chiquiar and Hanson, 2005; Ibarraran and Lubotsky, 2007; Fernández-Huertas Moraga, 2008; Villareal, 2014), although they could either be positively (e.g., Chiquiar and Hanson, 2005) or negatively selected (Ibarraran and Lubotsky, 2007; Fernández-Huertas Moraga, 2008; Villareal, 2014) based on earnings. In any event, our objective was not to focus on different incentives of the U.S. and Mexican social security systems but to complement our analysis of the determinants of retirement for Mexican-born immigrants in the United States by assessing other Mexican populations. As such, we believe that the possible self-selection of immigrants does not substantially affect our comparative findings.

Another limitation is that the HRS has no means to identify the documentation or legal status of Mexican-born immigrants. Indeed, to our knowledge, there is no study that identifies the undocumented population in the HRS. Undocumented immigrants often pay OASDI taxes, using illegitimate social security numbers, but social security benefits are not available to individuals without a work-authorized social security number (Aguila and Vega, 2017). The U.S. SSA has estimated that three-fourths of undocumented immigrants pay payroll taxes, contributing $6–$7 billion to social security funds that they are unable to claim (Porter, 2005). Previous studies for the United States using other data sets have also found that undocumented immigrants are undercounted (Capps *et al*., 2003; Passel and Cohn, 2010; Amuedo-Dorantes and Bansak, 2014; Bohn *et al*., 2014). We cannot assume that the Mexican immigrants without HRS-SSA linkage are undocumented. Some with missing linkages may have had their social security numbers misreported or miscoded (Olson, 1999).

Our inability to identify the legal status of Mexicans in the HRS, however, does not undermine our findings. To the extent that our sample of Mexican-born immigrants may have undocumented individuals is the extent to which we are underestimating retirement determinants. The inability of undocumented Mexican-born immigrants to claim social security benefits would mean specifically that we are underestimating the relevance of the social security wealth measure and peak value for retirement decisions. Because our estimates for Mexican-born immigrants aligned with previous research, we believe that the interpretation of our findings would not change with the possible inclusion of undocumented immigrants in the sample.

We were concerned that the small sample size for Mexican-born immigrants in the HRS would make our estimates imprecise. To assess the validity of our estimates, we conducted robustness tests with additional HRS waves (increasing the sample size of Mexican-born immigrants) and found that the increased sample provides better precision to our analysis but generates qualitatively similar estimates. Another potential issue is that the sample of Mexican-born immigrants was younger than the sample of non-Hispanic whites. In our original sample of Mexican-born immigrants, 9.6% were 65 years or older. The small sample size may prevent the identification of the association between retirement on the one hand and being older and Medicare-eligible on the other, both of which affect retirement. To evaluate the potential shortcoming, we increased our sample by including additional HRS waves – thereby increasing the proportion of individuals 65 years or older to 13.5% – and found qualitatively similar estimates. In fact, the Mexican-born population in our HRS sample, while having a different population structure than non-Hispanic whites in the United States, mirrored the age distribution of the Mexican origin individuals in Mexico. We consistently observed in both the HRS and MHAS a lower proportion of older adults of Mexican origin than among non-Hispanic whites. We observed that the non-migrants in MHAS were responsive to other drivers of retirement found in previous studies, but Mexican-born immigrants in HRS and return-migrants in MHAS were not.

## 6. Conclusions and policy implications

Mexican-origin workers are a significant population in the United States but also an economically vulnerable one. We built on previous research (e.g., Gruber and Wise, 2004) by illuminating the retirement determinants for lower-income immigrants. Mexican-born immigrants are, in general, less responsive to most sociodemographic and institutional (e.g., health insurance) drivers of retirement than are non-Hispanic whites; yet, they responded as non-Hispanic whites did to social security and private pension incentives.

Our results were consistent with previous research that found that Hispanics rely more than other groups on social security benefits to sustain their post-retirement years (Hendley and Bilimoria, 1999; Social Security Administration, 2010). Naturally, the social security benefits that comprise most retirement income for Mexican-born immigrants had a far greater effect on retirement decisions than did other sociodemographic, health, or institutional influences. Given the impact of social security wealth in our analyses, we expect future changes in the U.S. social security system will strongly affect labor force participation and retirement decisions of Mexican-born immigrants. This also may imply that income-assistance programs for older persons could greatly benefit and influence the retirement decisions of low-income older adults.

The lack of responsiveness by U.S. Mexican-born immigrants to several other determinants of retirement may also suggest unobserved preferences or lack of knowledge about how to support their retirement. Identifying such unobserved preferences, or areas of limited understanding, and their influence on retirement for migrating populations is beyond the scope of this paper but deserves further investigation and consideration by policymakers.

The lack of responsiveness to social security incentives for return-migrants might change with the introduction of a totalization agreement between the United States and Mexico. Totalization agreements allow workers to combine social security work credits from both countries, and thereby reach eligibility in either country more quickly. Instituting such an agreement would enable Mexican-born immigrants and return-migrants to more readily access social security benefits and to improve their wellbeing in retirement. The lack of a totalization agreement reduces incentives for Mexican-born workers in the United States to return to Mexico because (1) they could lose their contributions to the U.S. social security system and (2) the time they have spent in the United States does not count toward the 25 years of contributions they need to claim Mexican social security benefits.

## Supplementary Material

Online Supplementary file

## Figures and Tables

**Table 1.** Summary statistics for HRS and MHAS 2012

	HRS 2012		MHAS 2012	
	Non-Hispanic whites % or Mean (SD)	Mexican-born immigrants % or Mean (SD)	Non-migrants % or Mean (SD)	Return-migrants % or Mean (SD)
Retire in *t*	15.82	7.07	26.27	25.19
Covariates				
Age	61.88 (7.53)	57.99 (5.04)	60.38 (6.84)	62.75 (8.22)
Cohort 1	24.89	8.68	7.24	18.23
Cohort 2	14.72	3.22	19.02	18.80
Cohort 3	25.38	36.66	24.39	20.68
Cohort 4	35.01	51.44	30.58	26.88
Male	47.45	51.45	61.78	85.34
Years of education	14.08 (2.26)	7.65 (4.47)	6.84 (5.04)	5.79 (4.55)
Couple (1 = yes, 0 = no)	69.65	76.21	74.06	80.45
No. of household members	2.32 (1.08)	3.75 (1.90)	2.97 (2.12)	2.83 (1.95)
Real monthly salary income (USD – HRS; USD PPP – MHAS)	2,313.57 (1,645.18)	1,575.07 (891.18)	276.77 (508.99)	203.88 (469.89)
Real net wealth (USD – HRS; USD PPP – MHAS)	602,791.10 (1,397,970.03)	94,795.61 (183,936.60)	151,667.97 (228,468.50)	140,199.17 (184,024.79)
U.S. citizen/resident (1 = yes, 0 = no)	–	–	0.00	1.32
Chronic conditions (1 = yes, 0 = no)	60.33	49.84	45.99	45.11
CES-D score (0–8)	0.93 (1.57)	1.58 (2.12)	1.88 (2.03)	1.79 (2.01)
Medicare eligibility in U.S. (1 = 65 +, 0 = no)	33.01	8.36	–	–
Emp health insurance U.S. (1 = yes, 0 = no)	31.36	19.29	–	–
Health insurance social security MX (1 = yes, 0 = no)	–	–	54.26	47.74
Receives *Seguro Popular* (1 = yes, 0 = no)	–	–	30.58	35.71
Real annual out-of-pocket exp (USD – HRS; USD PPP – MHAS)	2,968.25 (4,942.93)	1,956.93 (3,465.05)	344.72 (1,988.41)	550.04 (4,510.88)
Eligible non-contributory pension MX (1 = 65 +, 0 = no)	–	–	27.29	39.29
Contributed private pensions in U.S.			–	–
Defined benefit plans (1 = yes, 0 = no)	25.14	11.58	–	–
Defined contribution plans (1 = yes, 0 = no)	37.23	15.43	–	–
Both (1 = yes, 0 = no)	3.65	2.57	–	–
None (1 = yes, 0 = no)	33.98	70.42	–	–
Contributed social security in MX (1 = yes, 0 = no)	–	–	65.44	64.10
Contributed social security in U.S. (1 = yes, 0 = no)	–	–	0.00	24.25
No. observations	3,648	311	3,990	532

*Notes:* Emp health insurance U.S. refers to employer-sponsored health insurance; PP to private pensions; SS to social security, USD to U.S. dollars; USD PPP to U.S. dollars purchasing power parity. Our sample included the following HRS cohorts: cohort 1 refers to those born before 1941 (reference), cohort 2 refers to those born 1942–1947, cohort 3 refers to those early baby boomers born 1948–1953, and cohort 4 refers to mid baby boomers born 1954–1959. Standard deviation (SD) in parenthesis.

*Source*: author’s calculations.

**Table 2.** Social security wealth and peak value (USD)

	Non-Hispanic white (HRS)		Mexican-born immigrants (HRS)	
Age	SSW 50th	PV 50th	SSW 50th	PV 50th
60	138,255	27,314	127,359	46,646
61	143,491	26,483	135,634	66,505
62	151,343	23,824	149,804	50,278
63	161,027	18,019	160,031	40,540
64	175,027	11,865	178,743	17,861
65	179,917	8,596	183,827	15,187
66	188,772	5,261	188,128	13,432
67	194,603	2,570	195,857	8,680
68	200,166	837	200,693	3,839
69	204,508	−10,599	206,613	−9,287
70	190,567	−11,029	193,202	−9,752

*Notes*: Percentiles were computed separately for each group. SSW refers to social security wealth; PV to peak value; USD to U.S. dollars.

*Source*: author’s calculations.

**Table 3.** Marginal effects of the probability of retirement for HRS respondents (2012–2014)

	Non-Hispanic whites (HRS)			Mexican-born immigrants (HRS)		
	Coef.	SE	p	Coef.	SE	p
Specification I: no controls						
Social security wealth (10,000)	0.011***	[0.001]	0.000	0.013***	[0.003]	0.000
Peak value (10,000)	−0.039***	[0.004]	0.000	−0.018*	[0.010]	0.091
Specification II: sociodemographic						
Social security wealth (10,000)	0.006***	[0.001]	0.000	0.009***	[0.004]	0.001
Peak value (10,000)	−0.008	[0.006]	0.209	−0.012	[0.013]	0.377
Cohort 1	0.167***	[0.024]	0.000	0.002	[0.045]	0.970
Cohort 2	0.117***	[0.023]	0.000	0.142*	[0.084]	0.092
Cohort 3	0.049***	[0.019]	0.009	0.040	[0.033]	0.223
Male	−0.001	[0.013]	0.915	0.036	[0.027]	0.176
Years of education	−0.011***	[0.003]	0.000	−0.005**	[0.003]	0.049
Couple (1 = yes, 0 = no)	−0.002	[0.014]	0.877	0.002	[0.035]	0.962
No. of household members	−0.002	[0.006]	0.791	−0.004	[0.007]	0.585
Ln monthly salary income	−0.019***	[0.005]	0.000	−0.018*	[0.010]	0.083
Real net wealth (100,000)	−0.001	[0.001]	0.137	0.001	[0.005]	0.793
Specification III: sociodemographic, health, health care, and private pensions						
Social security wealth (10,000)	0.005***	[0.002]	0.000	0.009**	[0.004]	0.014
Peak value (10,000)	−0.001	[0.008]	0.950	−0.013	[0.018]	0.464
Cohort 1	0.154**	[0.026]	0.000	0.015	[0.046]	0.737
Cohort 2	0.106***	[0.024]	0.000	0.184*	[0.096]	0.054
Cohort 3	0.050**	[0.019]	0.010	0.042	[0.042]	0.158
Male	−0.002	[0.013]	0.858	0.046*	[0.024]	0.059
Years of education	−0.009***	[0.003]	0.000	−0.002	[0.003]	0.390
Couple (1 = yes, 0 = no)	0.008	[0.014]	0.580	0.007	[0.032]	0.817
No. of household members	−0.001	[0.006]	0.865	−0.003	[0.006]	0.690
Ln monthly salary income	−0.014***	[0.005]	0.010	−0.015	[0.011]	0.145
Real net wealth (100,000)	−0.001	[0.001]	0.181	0.004	[0.006]	0.478
Chronic conditions (1 = yes, 0 = no)	0.036***	[0.012]	0.003	0.049*	[0.026]	0.056
CES-D score (0–8)	0.010***	[0.004]	0.005	0.010*	[0.005]	0.080
Medicare eligibility in US (1 = 65 +, 0 = no)	0.025	[0.026]	0.415	0.001	[0.061]	0.989
Emp health insurance US, (1 = yes, 0 = no)	0.003	[0.014]	0.841	−0.016	[0.037]	0.669
Ln annual out-of-pocket exp	0.002	[0.001]	0.210	−0.001	[0.002]	0.814
Contributed private pensions in U.S.						
Defined contribution plans	−0.061***	[0.017]	0.000	0.013	[0.032]	0.669
Both	−0.049	[0.014]	0.121	0.118	[0.121]	0.327
None	0.004	[0.032]	0.823	0.058**	[0.029]	0.043
No. observations	3,648			311		

*Notes*: Emp health insurance U.S. refers to employer-sponsored health insurance. Our sample included the following HRS cohorts: cohort 1 refers to respondents born before 1941, cohort 2 refers to respondents born 1942–1947, cohort 3 refers to early baby boomers born 1948–1953, and cohort 4 refers to mid baby boomers born 1954–1959 (reference). Social security wealth, peak value, and real net wealth are in U.S. dollars. Our reference category for Contributed to private pensions in U.S. is defined benefit plans. Standard errors (SE) in parentheses.

*Source*: author’s calculations.

* p < 0.1,

** p < 0.05,

*** p < 0.01.

**Table 4.** Marginal effects of the probability of retirement for HRS (2000–2014)

	Non-Hispanic whites (HRS)			Mexican-born immigrants (HRS)		
	Coef.	SE	p	Coef.	SE	p
Specification I: no controls						
Social security wealth (10,000)	0.014***	[0.001]	0.000	0.014***	[0.002]	0.000
Peak value (10,000)	−0.040***	[0.002]	0.000	−0.031***	[0.009]	0.001
Specification II: sociodemographic						
Social security wealth (10,000)	0.009***	[0.001]	0.000	0.015***	[0.003]	0.000
Peak value (10,000)	−0.013***	[0.003]	0.000	−0.033***	[0.012]	0.006
Cohort 1	0.129***	[0.010]	0.000	−0.041	[0.045]	0.364
Cohort 2	0.089***	[0.010]	0.000	−0.010	[0.049]	0.841
Cohort 3	0.035***	[0.008]	0.000	−0.012	[0.031]	0.711
Male	0.001	[0.005]	0.990	0.007	[0.019]	0.708
Years of education	−0.010***	[0.001]	0.000	−0.004*	[0.002]	0.067
Couple (1 = yes, 0 = no)	−0.004	[0.006]	0.492	−0.003	[0.023]	0.904
No. of household members	−0.008***	[0.003]	0.003	−0.003	[0.006]	0.567
Ln monthly salary income	−0.021***	[0.002]	0.000	−0.018**	[0.009]	0.033
Real net wealth (100,000)	−0.001**	[0.001]	0.014	0.005	[0.003]	0.111
Specification III: sociodemographic, health, health care, and private pensions						
Social security wealth (10,000)	0.007***	[0.001]	0.000	0.011***	[0.003]	0.001
Peak value (10,000)	−0.003	[0.005]	0.445	−0.011	[0.015]	0.440
Cohort 1	0.123***	[0.010]	0.000	−0.053	[0.043]	0.215
Cohort 2	0.091***	[0.010]	0.000	0.018	[0.052]	0.728
Cohort 3	0.037***	[0.008]	0.000	−0.009	[0.030]	0.753
Male	−0.001	[0.005]	0.911	0.022	[0.018]	0.242
Years of education	−0.008***	[0.001]	0.000	−0.003	[0.002]	0.212
Couple (1 = yes, 0 = no)	0.003	[0.006]	0.638	0.001	[0.022]	0.961
No. of household members	−0.008***	[0.003]	0.003	−0.002	[0.005]	0.652
Ln monthly salary income	−0.013***	[0.002]	0.000	−0.014	[0.009]	0.126
Real net wealth (100,000)	−0.001**	[0.001]	0.041	0.005*	[0.003]	0.060
Chronic conditions (1 = yes, 0 = no)	0.034***	[0.005]	0.000	0.041**	[0.017]	0.013
CES-D score (0–8)	0.009***	[0.002]	0.000	0.005	[0.003]	0.135
Medicare eligibility in US (1 = 65 +, 0 = no)	0.017*	[0.010]	0.095	0.084	[0.051]	0.100
Emp health insurance U.S. (1 = yes, 0=no)	−0.022***	[0.006]	0.000	−0.032	[0.021]	0.133
Ln annual out-of-pocket exp	−0.001	[0.001]	0.370	0.001	[0.001]	0.819
Contributed private pensions in U.S.						
Defined contribution plans	−0.053***	[0.006]	0.000	−0.058**	[0.028]	0.036
Both	−0.013	[0.018]	0.486	0.020	[0.079]	0.805
None	−0.021***	[0.007]	0.003	0.003	[0.030]	0.923
No. observations	22,615			1,033		

*Notes:* Emp health insurance U.S. refers to employer-sponsored health insurance. Our sample included the following HRS cohorts: cohort 1 refers to respondents born before 1941, cohort 2 refers to respondents born 1942–1947, cohort 3 refers to early baby boomers born 1948–1953, and cohort 4 refers to mid baby boomers born 1954–1959 (reference). Social security wealth, peak value, and real net wealth are in U.S. dollars. Year fixed-effects are included. Our reference category for Contributed to private pensions in U.S. is defined benefit plans. Standard errors (SE) in parentheses.

*Source*: author’s calculations.

* p < 0.1,

** p < 0.05,

*** p < 0.01.

**Table 5.** Marginal effects of the probability of retirement for MHAS samples of non-migrants and return-migrants

	Non-migrants			Return-igrants		
	Coef.	SE	p	Coef.	SE	p
Cohort 1	0.092*	[0.051]	0.071	0.082	[0.103]	0.426
Cohort 2	0.000	[0.047]	0.999	0.051	[0.099]	0.606
Cohort 3	0.087***	[0.017]	0.000	0.026	[0.050]	0.611
Male	−0.169***	[0.015]	0.000	−0.195***	[0.053]	0.000
Years of education	−0.001	[0.002]	0.378	−0.004	[0.005]	0.409
Couple (1 = yes, 0 = no)	0.028*	[0.016]	0.091	−0.013	[0.049]	0.791
No. of household members	0.009***	[0.003]	0.002	0.018**	[0.009]	0.035
Ln monthly salary income	−0.013***	[0.002]	0.000	0.001	[0.006]	0.849
Real net wealth (100,000)	−0.003	[0.003]	0.323	−0.006	[0.009]	0.551
US citizen/resident (1 = yes, 0 = no)	–	–	–	0.016	[0.148]	0.916
Chronic conditions (1 = one or more, 0 = none)	0.069***	[0.013]	0.000	0.046	[0.036]	0.194
CES-D score (0–8)	0.008**	[0.003]	0.028	0.006	[0.009]	0.525
Health insurance social security MX (1 = yes, 0 = no)	0.039*	[0.023]	0.088	0.080	[0.059]	0.172
Receives *Seguro Popular* (1 = yes, 0 = no)	−0.030	[0.026]	0.259	−0.003	[0.069]	0.966
Ln annual out-of-pocket exp	−0.001	[0.001]	0.355	0.001	[0.002]	0.660
Eligible to non-contributory pension in MX (1 = 65+, 0 = no)	0.146***	[0.046]	0.002	0.122	[0.093]	0.193
Contributed Social Security in MX (1 = yes, 0 = no)	0.037**	[0.018]	0.035	−0.001	[0.046]	0.982
Contributed Social Security in U.S. (1 = yes, 0 = no)	–	–	–	−0.037	[0.043]	0.390
No. Observations	3,990			532		

*Notes*: Our sample included the following cohorts: cohort 1 refers to respondents born before 1941, cohort 2 refers to respondents born 1942–1947, cohort 3 refers to respondents born between 1948 and 1953, and cohort 4 refers to respondents born between 1954 and 1959 (reference). Real net wealth is in U.S. dollars purchasing power parity. Standard errors (SE) in parentheses.

*Source*: author’s calculations.

* p < 0.1,

** p < 0.05,

*** p < 0.01.

## Footnotes

1 Because employers bear more costs and more ‘accrual risks’ in offering DB plans, and because the Pension Protection Act of 2006 requires employers to fully pre-fund the plans, many employers have switched from DB to DC plans (Martin and Rafsky, 2007).

2 Self-employed workers in the United States are also required to report their labor earnings for social security on their income tax returns (Zissimopoulos *et al*., 2007). Nevertheless, there are exceptions of employees not covered by social security: (1) state, country, or municipal employees who are covered by state-funded pension plans instead of social security, (2) government employees hired before 1984, who receive pensions under the old Civil Service Retirement System, (3) railroad employees, covered by their own pension system since the 1930s, and (4) foreign nationals working in the United States for their home governments or international organizations (AARP, 2021). Additionally, there may be cases of noncompliance with the law. For example, some of the self-employed may evade social security contributions by reporting capital earnings instead of labor earnings.

3 While the benefit calculations of the social security could vary by location due to heterogeneous costs of living, social security benefits are homogeneous across locations.

4 The informal sector includes self-employed workers who may choose not to contribute to the social security system as well as voluntary and involuntary salaried workers. Most salaried workers in the informal sector are involuntary. These workers typically have few skills or work for small firms. Firms in the informal sector typically have fewer than five employees with little potential to grow or are mid-size non-compliant firms with some of their employees off the books (Perry et al., 2007).

5 The SSA records include detailed earnings (both those covered and those not covered by social security), covered self-employment earnings, and earnings caps for each year.

6 The samples of non-migrants and return-migrants included individuals in the formal and informal sectors. Some individuals may spend all their working lives in the formal or informal sectors, but a large proportion of the labor force spend their working life in both sectors (Cox and Rodriguez-Oreggia, 2006). We did not want to exclude the group that spent their working life in both sectors, so we decided to include all workers among non-migrants and return-migrants in our analytic sample.

7 Because the prospects of losing employer-sponsored health insurance coverage can influence the timing of retirement in the United States (Cutler and Zeckhauser, 1999; Nyce et al., 2013), we controlled for such access in our analysis.

8 The PIA is the benefit an individual would receive should she choose to retire and begin receiving retirement benefits at her full retirement age.

9 The PIA is calculated to be a sum of (1) 90 percent of the earnings up to the first threshold or ‘bend-point’, (2) 32 percent of the remaining earnings, up to the second threshold, and (3) 15 percent of the remainder. The full list of thresholds or bend-points that vary by year can be found at the social security Administration webpage (Social Security Administration, n.d.a, n.d.b).

10 Knapp (2014) found that both the spousal and survivor benefits have only a small effect on household labor supply decisions by primary earners and their spouses.

11 In this study, we used the real discount rate of 3% following previous literature (e.g. Börsch-Supan, 1992; Coile and Gruber, 2000, 2001).

12 Following Coile and Gruber (2007), we assumed that, (1) individuals retiring before age 62 delay claiming until age 62 but not later, and (2) individuals retiring at age 62 or later claim social security benefits immediately.

13 In an option value model (Stock and Wise, 1990), an employee would compare the expected present value of retiring at the current age with the value of retiring at each subsequent age. This process assumes workers’ utility maximization tendencies whereby an employee weights the indirect utility of future income to that of future retirement benefits. The option value of postponing retirement is equal to the maximum of the present value of retiring at each future age minus the expected present value of immediate retirement. Previous studies have shown that the option value model approximates workers’ retirement decisions as well as a more complex dynamic programming model (e.g. Lumsdaine et al., 1992; Ausink and Wise, 1996).

14 The monthly benefit level reflects the primary insurance amount (PIA) reduced for claiming early or increased for delaying claiming benefits past the full retirement age.

15 We also pooled the 2012–2014 HRS sample and the 2000–2014 HRS sample of non-Hispanic Whites and Mexican-born immigrants and interacted the dummy variable for Mexican-born immigrants with all covariates to compare differences between groups (see results in the online Appendix). We ran the models with OLS because the straightforward interpretation of interaction terms of linear models changes in non-linear models (Ai and Norton, 2003). We used results of the interactions terms of the covariates with the Mexican-born immigrant indicator to describe differences across groups.

16 Among such studies is one conducted by Gustman and Steinmeier (2000), who found that *high*-earnings immigrants with up to 20 years in the United States benefited more than the U.S.-born from claiming U.S. social security benefits. The authors noted that the social security benefit formula benefits workers with few lifetime covered earnings and that the years before their arrival to the United States were considered as zero earnings. This, in turn, ultimately increased the replacement rate for such workers, who had higher average earnings than the native-born. Borjas (2011) showed that the ten-year work rule for social security benefits eligibility had a substantial effect on newly arrived older immigrants’ labor supply around retirement age. The author found that older immigrants typically continue working until they complied with the minimum ten years needed to qualify for social security, after which their probability of employment dropped.
