# Supplementary material for: Migration, work, and retirement: the case of Mexican-origin populations
Source: J Pension Econ Financ. Author manuscript; Available in PMC 2023 May 12. (PMC10181811; doi:10.1017/s1474747221000342)
Supplement: Online Supplementary file [file NIHMS1825937-supplement-Online_Supplementary_file.docx]

**Online Appendix**

**Migration, Work, and Retirement: The Case of Mexican-Origin Populations**

**Health and Retirement Study (HRS) Sample**

**Figure A1. Study Sample**

Dropped 26 (3.1%)

no follow-up in 2014 n=348

Dropped 37 (4.4%) missing in variables

n=311

cv

174 with

imputed earnings

2012 HRS

n= 12,755

841 (6.6%)

Mexican-born

11,914 (93.4%)

non-Hispanic whites

Dropped 49 (5.8%)

outside the age range 50-80

n=792

Dropped 2,305 (19.3%)

outside the age range 50-80

n=9,609

1,134 with

imputed earnings

2,514 with

HRS-SSA link

137 with

HRS-SSA link

n=311

n=3,648

Dropped 418 (49.7%)

not working in 2012

n=374

cv

Dropped 5,499 (46.2%)

not working in 2012

n=4,110

Dropped 306 (2.6%)

no follow-up in 2014 n=3,804

Dropped 156 (1.3%) missing in variables

n=3,648

412 (49.0%)

imputed earnings

2,743 (23.0%)

imputed earnings

9,171 (77.0%)

HRS-SSA link

429 (51.0%)

HRS-SSA link

The 2012 Health and Retirement Study (HRS) includes 841 Mexican-born and 11,914 U.S.-born non-Hispanic white individuals, as shown in Figure A1. Among the 841 Mexican-born individuals, 429 have earnings history through linkages to U.S. Social Security Administration (SSA) data, and for 412 we imputed past earnings (see imputation method described below). Among the 11,914 non-Hispanic whites, 9,171 have earnings history through HRS-SSA linkages, and for 2,743 we imputed past earnings. We excluded from our analysis 49 (5.8%) Mexican-born and 2,305 (19.3%) non-Hispanic White respondents for being outside our analysis age group—between ages 50 and 80 in 2012. We then dropped 418 of the Mexican-born individuals (49.7% of the original sample of 841) and 5,499 (46.2% of the original sample of 11,914) of the U.S.-born non-Hispanic white individuals because they did not work in 2012. We removed 26 (3.1% of the original sample of 841) Mexican-born and 306 (2.6% of the original sample of 11,914) non-Hispanic White respondents with no follow-up interview in 2014. Finally, we dropped 37 (4.4% of the original sample) Mexican-born and 156 (1.3% of the original sample) non-Hispanic whites because they have missing covariates. That left us with a sample of 311 Mexican-born individuals (137 with HRS-SSA linked earnings and 174 with imputed earnings) and 3,648 non-Hispanic whites (2,514 with HRS-SSA linked earnings and 1,134 with imputed earnings).

**MHAS Sample**

In this study, we analyze Mexican return migrants and non-migrants. The Mexican Health and Aging Study (MHAS) survey for 2012 includes 15,723 respondents. Of these, 1,562 were return migrants and 14,161 were non-migrants (see Figure A2). We excluded from our analysis 233 (14.9% of the original sample of 1,562) return migrants and 1,871 (13.2% of the original sample of 14,161) non-migrant respondents for being outside our analysis age group of 50 to 80 years old in 2012. We then dropped 665 return migrants (42.6% of the original sample) and 7,463 non-migrants (52.7% of the original sample) because they did not work in 2012. We removed 87 (5.6% of the original sample) return migrants and 628 (4.4% of the original sample) non-migrants with no follow-up interview in 2015. Finally, we dropped 45 (2.9% of the original sample) return migrants and 209 (1.5% of the original sample) non-migrants because they have missing covariates. This yielded analytical samples of 3,990 non-migrant respondents and 532 return-migrants.

**Figure A2. Sample MHAS 2012 Return Migrants and Non-migrants**

Dropped 87 (5.6%)

no follow-up in 2015 n=577

Dropped 45 (2.9%)

missing in variables

n=532

cv

2012 MHAS

n= 15,723

1,562 (9.9%)

Return migrants

14,161 (90.1%)

Non-migrants

Dropped 233 (14.9%)

outside the age range 50-80

n=1,329

Dropped 1,871 (13.2%)

outside the age range 50-80

n=12,290

n=532

n=3,990

Dropped 665 (42.6%)

not working in 2012

n=664

cv

Dropped 7,463 (52.7%)

not working in 2012

n=4,827

Dropped 628 (4.4%)

no follow-up in 2015 n=4,199

Dropped 209 (1.5%)

missing in variables

n=3,990

**Approximation of Future HRS Earnings (Projecting to Age 120)**

For 2015 and subsequent years, we assume surviving HRS respondents will see their earnings increase 1 percent each year.

**Imputations of Past HRS Earnings**

For person-year observations showing respondents working but having zero or negative earnings, as well as for person-year observations with missing HRS-SSA linkages, we imputed the past earnings. We used a multiple imputation technique that involves iterative stochastic imputation strategies, available as a pre-written STATA package. This method allows for imputation of zeroes and imputation of earnings brackets used in the surveys to recover nonresponse (Wong and Espinoza, 2004). We used age, sex, race, cohort, and education covariates. In the multiple imputation technique, overall distributions of the observed data are used to estimate multiple imputation values, accounting for the uncertainty around the true earnings value that is missing (Johnson and Young, 2011; White et al., 2011). We generated five multiple imputations of earnings for each person-year observation, and set the median of the five imputations as the value to be used for estimating individuals’ Social Security wealth (Lokupitiya et al., 2006; Ni et al., 2005; White et al., 2018). If the imputed value of earnings was (a) equal to or below zero or (b) above the 95^th^ percentile of the unimputed earnings distribution in the SSA-HRS linked records for each year, we re-imputed the earnings. The re-imputation process was repeated 22 times. Our ultimate earnings distribution was similar to that reported for those with HRS-SSA linked earnings.

**ACS and HRS samples of Mexican-born Immigrants**

We compare the sample of Mexican-born immigrants between ages 50 and 80 in 2012 in the American Community Survey (ACS) and the HRS (Table A1). We find that the samples are comparable in household size and educational attainment. We observe that mean age was slightly higher and the proportion of males and of respondents in couples slightly lower in the ACS than the HRS.

| **Table A1 Summary Statistics of the Mexican-born in the HRS and in the ACS in 2012** | | | |
| --- | --- | --- | --- |
|  | ACS 2012 | HRS 2012 | Chi-square/t-test |
|  | % or Mean | % or Mean | p-value |
| Male | 49.241 | 52.035 | p<0.001*** |
|  | (0.499) | (0.500) |  |
| Age | 59.931 | 57.916 | p<0.001*** |
|  | (7.916) | (5.018) |  |
| Household size | 3.95 | 3.811 | p>0.1 |
|  | (2.127) | (1.965) |  |
| Couple (1=yes, 0=no) | 69.48 | 76.71 | p<0.001*** |
|  | (0.460) | (0.423) |  |
| Educational Attainment | 7.679 | 7.584 | p>0.1 |
|  | (5.018) | (4.419) |  |
| Real monthly salary income (USD) | 1648.059 | 1,562.39 | p<0.1* |
|  | (2087.437) | (881.832) |  |
| N | 16,356 | 344 |  |
| *Notes*: Monthly salary income is converted to 2012 U.S. dollars. **p<0.1 **p<0.05 ***p<0.01*  Source: HRS 2012 and ACS 2012. ACS 1-year data available at: <https://www.census.gov/programs-surveys/acs/microdata/access.2012.html> | | | |

Overall, we find the characteristics of our HRS sample are similar to those of the comparable ACS sample. Both samples are younger than the comparable non-Hispanic white population, and also have lower levels of educational attainment.

**Statistical Differences Between Mexican-born Immigrants and Non-Hispanic White**

We pooled the sample of non-Hispanic Whites and Mexican-born immigrants and interacted the group identifier with the covariates to assess differences among groups. We ran the model with OLS because the interpretation of interaction terms with a Probit model is less straightforward (Ai and Norton, 2003). The results for the 2012-2014 and for the 2000-2014 samples are shown in Table A2-1 for Specification I and II and in Table A2-2 for Specification III. We describe differences among non-Hispanic white and Mexican-born in the main article when discussing the results in Tables 3 and 4 using the results of the interaction terms between covariates and the Mexican-born indicator in Tables A2-1 and A2-2.

| **Table A2-1. OLS Effects of the Probability of Retirement for HRS Pooled Samples, Specification I and II** | | | | | | | | | |
| --- | --- | --- | --- | --- | --- | --- | --- | --- | --- |
|  | Sample 2012-2014 | | | | Sample 2000-2014 | | | | |
|  | Coef. | SE | *P* | Coef. | | SE | | *P* | |
| ***Specification I: No Controls*** |  |  |  |  | | |  | |  |
| Mexican-born (1=yes, 0=non-Hispanic white) | -0.058*** | [0.008] | 0.000 | -0.034*** | | | [0.007] | | 0.000 |
| Social Security Wealth (10,000) | 0.012*** | [0.001] | 0.000 | 0.014*** | | | [0.001] | | 0.000 |
| Social Security Wealth x Mexican-born | 0.005 | [0.004] | 0.236 | 0.005* | | | [0.003] | | 0.099 |
| Peak Value (10,000) | -0.047*** | [0.005] | 0.000 | -0.046*** | | | [0.003] | | 0.000 |
| Peak Value x Mexican-born | 0.013 | [0.022] | 0.548 | -0.011 | | | [0.019] | | 0.552 |
| ***Specification II: Sociodemographic*** |  |  |  |  | | |  | |  |
| Mexican-born (1=yes, 0=non-Hispanic white) | -0.353** | [0.165] | 0.033 | -0.250*** | | | [0.095] | | 0.009 |
| Social Security Wealth (10,000) | 0.006*** | [0.001] | 0.000 | 0.009*** | | | [0.001] | | 0.000 |
| Social Security Wealth x Mexican-born | 0.010** | [0.005] | 0.041 | 0.009** | | | [0.004] | | 0.012 |
| Peak Value (10,000) | -0.012* | [0.007] | 0.099 | -0.024*** | | | [0.004] | | 0.000 |
| Peak Value x Mexican-born | -0.025 | [0.024] | 0.300 | -0.031* | | | [0.019] | | 0.092 |
| Cohort 1 | 0.152*** | [0.025] | 0.000 | 0.091*** | | | [0.012] | | 0.000 |
| Cohort 1 x Mexican-born | -0.226*** | [0.074] | 0.002 | -0.095*** | | | [0.036] | | 0.008 |
| Cohort 2 | 0.101*** | [0.025] | 0.000 | 0.056*** | | | [0.010] | | 0.000 |
| Cohort 2 x Mexican-born | 0.060 | [0.107] | 0.576 | -0.024 | | | [0.043] | | 0.574 |
| Cohort 3 | 0.028 | [0.018] | 0.120 | 0.014* | | | [0.007] | | 0.055 |
| Cohort 3 x Mexican-born | -0.011 | [0.032] | 0.720 | -0.011 | | | [0.014] | | 0.426 |
| Male | -0.003 | [0.012] | 0.791 | -0.003 | | | [0.005] | | 0.577 |
| Male x Mexican-born | 0.045 | [0.029] | 0.117 | 0.024 | | | [0.018] | | 0.170 |
| Years of education | -0.011*** | [0.003] | 0.000 | -0.011*** | | | [0.001] | | 0.000 |
| Years of education x Mexican-born | 0.006 | [0.004] | 0.113 | 0.008*** | | | [0.003] | | 0.002 |
| Couple (1=yes, 0=no) | -0.003 | [0.014] | 0.806 | -0.006 | | | [0.006] | | 0.325 |
| Couple x Mexican-born | 0.018 | [0.034] | 0.612 | 0.010 | | | [0.023] | | 0.662 |
| No. of household members | -0.001 | [0.005] | 0.971 | -0.006** | | | [0.002] | | 0.015 |
| No. household members x Mexican-born | -0.003 | [0.009] | 0.703 | -0.001 | | | [0.006] | | 0.907 |
| Ln monthly salary income | -0.022*** | [0.007] | 0.001 | -0.024*** | | | [0.003] | | 0.000 |
| Ln monthly salary income x Mexican-born | -0.010 | [0.020] | 0.631 | -0.003 | | | [0.013] | | 0.814 |
| Real net wealth (100,000) | -0.001** | [0.001] | 0.041 | -0.001*** | | | [0.001] | | 0.000 |
| Real net wealth x Mexican-born | 0.003 | [0.009] | 0.741 | 0.005 | | | [0.004] | | 0.247 |
| No. observations | 3,959 |  |  | 3,959 | | |  | |  |
| Notes: Social security wealth, peak value, and real net wealth are in U.S. dollars. Our sample includes the following HRS cohorts: cohort 1 refers to those born before 1941, cohort 2 refers to those born 1942-1947, cohort 3 refers to those early baby boomers born 1948-1953, and cohort 4 refers to mid baby boomers born 1954-1959 (reference). Standard errors (SE) are in parentheses. Our reference category for pension is those with defined benefit plans. * p<0.1 ** p<0.05 *** p<0.01. Source: author’s calculations. | | | | | | | | | |

| **Table A2-2. OLS Effects of the Probability of Retirement for HRS Pooled Samples, Specification III** | | | | | | | | | | |  |
| --- | --- | --- | --- | --- | --- | --- | --- | --- | --- | --- | --- |
|  | Sample 2012-2014 | | | | | Sample 2000-2014 | | | | |  |
|  | | Coef. | SE | *P* | | Coef. | | SE | | *P* |  |
| ***Specification III: Sociodemographic, Health, Health care, and Private Pensions*** | | | | | | | | | | |  |
| Mexican-born (1=yes, 0=non-Hispanic white) | | -0.305** | [0.174] | 0.080 | -0.239** | | [0.095] | | 0.012 | | |
| Social Security Wealth (10,000) | | 0.005*** | [0.002] | 0.005 | 0.007*** | | [0.001] | | 0.000 | | |
| Social Security Wealth x Mexican-born | | 0.009 | [0.009] | 0.144 | 0.002 | | [0.005] | | 0.705 | | |
| Peak Value (10,000) | | -0.002 | [0.010] | 0.853 | -0.012** | | [0.006] | | 0.026 | | |
| Peak Value x Mexican-born | | -0.032 | [0.030] | 0.285 | 0.002 | | [0.025] | | 0.938 | | |
| Cohort 1 | | 0.134*** | [0.028] | 0.000 | 0.084*** | | [0.012] | | 0.000 | | |
| Cohort 1 x Mexican-born | | -0.188** | [0.087] | 0.031 | -0.103*** | | [0.036] | | 0.004 | | |
| Cohort 2 | | 0.084*** | [0.027] | 0.002 | 0.056*** | | [0.010] | | 0.000 | | |
| Cohort 2 x Mexican-born | | 0.093 | [0.111] | 0.404 | -0.003 | | [0.044] | | 0.943 | | |
| Cohort 3 | | 0.029 | [0.018] | 0.113 | 0.017** | | [0.008] | | 0.028 | | |
| Cohort 3 x Mexican-born | | 0.001 | [0.035] | 0.967 | -0.002 | | [0.014] | | 0.874 | | |
| Male | | -0.003 | [0.012] | 0.795 | -0.004 | | [0.005] | | 0.490 | | |
| Male x Mexican-born | | 0.058*** | [0.030] | 0.050 | 0.039** | | [0.018] | | 0.034 | | |
| Years of education | | -0.010*** | [0.003] | 0.001 | -0.009*** | | [0.002] | | 0.000 | | |
| Years of education x Mexican-born | | 0.006 | [0.004] | 0.133 | 0.007** | | [0.003] | | 0.011 | | |
| Couple (1=yes, 0=no) | | 0.008 | [0.014] | 0.596 | 0.001 | | [0.006] | | 0.849 | | |
| Couple x Mexican-born | | 0.010 | [0.034] | 0.761 | 0.010 | | [0.023] | | 0.675 | | |
| No. of household members | | -0.001 | [0.005] | 0.939 | -0.006** | | [0.002] | | 0.012 | | |
| No. of household members x Mexican-born | | -0.004 | [0.008] | 0.597 | 0.001 | | [0.006] | | 0.814 | | |
| Ln monthly salary income | | -0.017** | [0.007] | 0.016 | -0.016*** | | [0.003] | | 0.000 | | |
| Ln monthly salary income x Mexican-born | | -0.011 | [0.020] | 0.577 | -0.010 | | [0.013] | | 0.506 | | |
| Real net wealth (100,000) | | -0.001* | [0.001] | 0.054 | -0.001*** | | [0.001] | | 0.001 | | |
| Real net wealth x Mexican-born | | 0.006 | [0.009] | 0.514 | 0.006 | | [0.004] | | 0.182 | | |
| Chronic Conditions (1=yes, 0=no) | | 0.034*** | [0.012] | 0.003 | 0.031*** | | [0.005] | | 0.000 | | |
| Chronic Conditions x Mexican-born | | 0.018 | [0.033] | 0.580 | 0.011 | | [0.019] | | 0.571 | | |
| CES-D score (0-8) | | 0.010** | [0.004] | 0.013 | 0.009*** | | [0.002] | | 0.000 | | |
| CES-D x Mexican-born | | -0.002 | [0.008] | 0.797 | -0.004 | | [0.004] | | 0.317 | | |
| Medicare Eligibility in US (1=65+, 0=no) | | 0.043 | [0.039] | 0.267 | 0.023* | | [0.013] | | 0.077 | | |
| Medicare Eligibility in US x Mexican-born | | -0.030 | [0.160] | 0.850 | 0.123* | | [0.072] | | 0.087 | | |
| Emp health insurance US, (1=yes, 0=no) | | 0.009 | [0.012] | 0.468 | -0.015*** | | [0.005] | | 0.003 | | |
| Emp health insurance US x Mexican-born | | -0.025 | [0.037] | 0.505 | -0.006 | | [0.021] | | 0.757 | | |
| Ln annual out-of-pocket exp | | 0.001 | [0.001] | 0.253 | -0.001 | | [0.001] | | 0.471 | | |
| Ln annual out-of-pocket exp x Mexican-born | | -0.002 | [0.002] | 0.410 | 0.003 | | [0.001] | | 0.835 | | |
| Contributed Private Pensions in US | |  |  |  |  | |  | |  | | |
| Defined contribution plans | | -0.007 | [0.018] | 0.695 | -0.024*** | | [0.007] | | 0.000 | | |
| Defined contribution plans x Mexican-born | | -0.061 | [0.042] | 0.146 | 0.025 | | [0.037] | | 0.507 | | |
| Both | | -0.063*** | [0.015] | 0.000 | -0.073*** | | [0.006] | | 0.000 | | |
| Both x Mexican-born | | 0.023 | [0.034] | 0.494 | 0.024 | | [0.021] | | 0.268 | | |
| None | | -0.048* | [0.029] | 0.094 | -0.037** | | [0.016] | | 0.023 | | |
| None x Mexican-born | | 0.111 | [0.113] | 0.327 | 0.061 | | [0.052] | | 0.243 | | |
| No. observations | | 3,959 |  |  | 3,959 | |  | |  | | |
| Notes: Social security wealth, peak value, and real net wealth are in U.S. dollars. Our sample includes the following HRS cohorts: cohort 1 refers to those born before 1941, cohort 2 refers to those born 1942-1947, cohort 3 refers to those early baby boomers born 1948-1953, and cohort 4 refers to mid baby boomers born 1954-1959 (reference). Our reference category for pension is those with defined benefit plans. Standard errors (SE) are in parentheses. * p<0.1 ** p<0.05 *** p<0.01. Source: author’s calculations. | | | | | | | | | | |  |

**References**

Ai, C., and Norton, E. C. (2003). Interaction terms in logit and probit models. *Economics Letters*, *80*(1), 123–129. https://doi.org/10.1016/S0165-1765(03)00032-6

Johnson, D. R., and Young, R. (2011). Towards Best Practices in analyzing Datasets with Missing Data: Comparisons and Recommendations. *Journal of Marriage and Family*, *73*(5), 926–945. https://doi.org/10.2307/41329640

Lokupitiya, R., Lokupitiya, E., and Paustian, K. (2006). Comparison of missing value imputation methods for crop yield data. *Environmetrics*, *17*(4), 339–349. https://doi.org/10.1002/env.773

Ni, D., Leonard II, J. D., Guin, A., and Feng, C. (2005). Multiple imputation scheme for overcoming the missing values and variability issues in ITS data. *Journal of Transportation Engineering*, *131*(12), 931–938. https://doi.org/10.1061/(ASCE)0733-947X(2005)131:12(931)

White, I. R., Royston, P., and Wood, A. M. (2011). Multiple imputation using chained equations: Issues and guidance for practice. *Statistics in Medicine*, *30*(4), 377–399. https://doi.org/10.1002/sim.4067

White, K., Reiter, J. P., and Petrin, A. (2018). Imputation in US manufacturing data and its implications for productivity dispersion. *The Review of Economics and Statistics*, *100*(3), 502–509. https://doi.org/10.1162/REST_a_00678

Wong, R., and Espinoza, M. (2004). *Imputation of non-response on Economic Variables in the Mexican Health and Aging Study, MHAS. MHAS, 2001* (p. 84) [Project]. Mexican Health and Aging Study. url: http://goo.gl/59ack7
